# Further evidence that paroxysmal nocturnal haemoglobinuria is a disorder of defective cell membrane lipid rafts

**DOI:** 10.1111/jcmm.12605

**Published:** 2015-05-29

**Authors:** Mariusz Z Ratajczak, Sylwia Borkowska, Kasia Mierzejewska, Magda Kucia, Ewa Mendek-Czajkowska, Malwina Suszynska, Vivek A Sharma, Andrzej Deptala, Wechao Song, Uwe Platzbecker, Loree Larratt, Anna Janowska-Wieczorek, Jarek Maciejewski, Janina Ratajczak

**Affiliations:** a Stem Cell Institute at James Graham Brown Cancer Center, University of LouisvilleLouisville, KY, USA; b Department of Regenerative Medicine, Medical University of WarsawWarsaw, Poland; c Department of Physiology, Pomeranian Medical UniversitySzczecin, Poland; d Central Clinical Hospital MSW, Poland and Medical University of WarsawWarsaw, Poland; e Department of Medicine, University of PennsylvaniaPhiladelphia, PA, USA; f Universitaetsklinikum Carl-Gustav-CarusDresden, Germany; g Department of Medicine, University of AlbertaEdmonton, AB, Canada; h Tausig Cancer Center, Cleveland ClinicCleveland, OH, USA

**Keywords:** PNH, complement, CXCR4, S1P, lipid rafts

## Abstract

The glycolipid glycosylphosphatidylinositol anchor (GPI-A) plays an important role in lipid raft formation, which is required for proper expression on the cell surface of two inhibitors of the complement cascade, CD55 and CD59. The absence of these markers from the surface of blood cells, including erythrocytes, makes the cells susceptible to complement lysis, as seen in patients suffering from paroxysmal nocturnal haemoglobinuria (PNH). However, the explanation for why PNH-affected hematopoietic stem/progenitor cells (HSPCs) expand over time in BM is still unclear. Here, we propose an explanation for this phenomenon and provide evidence that a defect in lipid raft formation in HSPCs leads to defective CXCR4- and VLA-4-mediated retention of these cells in BM. In support of this possibility, BM-isolated CD34^+^ cells from PNH patients show a defect in the incorporation of CXCR4 and VLA-4 into membrane lipid rafts, respond weakly to SDF-1 stimulation, and show defective adhesion to fibronectin. Similar data were obtained with the GPI-A^−^ Jurkat cell line. Moreover, we also report that chimeric mice transplanted with CD55^−/−^ CD59^−/−^ BM cells but with proper GPI-A expression do not expand over time in transplanted hosts. On the basis of these findings, we propose that a defect in lipid raft formation in PNH-mutated HSPCs makes these cells more mobile, so that they expand and out-compete normal HSPCs from their BM niches over time.

## Introduction

Hematopoietic stem/progenitor cells (HSPCs) are retained in bone marrow (BM) niches because of the stromal-derived growth factor 1 (SDF-1)–CXCR4 receptor axis and very late antigen 4 (VLA-4 or α_4_β_1_ integrin)–vascular adhesion molecule 1 (VCAM-1 or CD106) interaction 1–4. While HSPCs express CXCR4 and VLA-4, their corresponding ligands, SDF-1 and VCAM-1, are expressed by cells in the BM microenvironment (*e.g*. osteoblasts and fibroblasts). It has been proposed that retention of HSPCs in BM is an active process that counteracts the sphingosine-1-phosphate (S1P) chemotactic gradient present in peripheral blood (PB) and that both CXCR4 and VLA-4 are lipid raft-associated receptors 5–8. In support of this model, administration of small molecule antagonists of CXCR4 and VLA-4 results in attenuation of HSPC retention in BM and egress of HSPCs from BM into PB in an S1P-dependent manner 5–8.

Paroxysmal nocturnal haemoglobinuria (PNH) is an acquired haemolytic anaemia and stem cell disease because of somatic mutation in PIG-A gene responsible for glycosylphosphatidylinositol anchor (GPI-A) biosynthesis 9–12. GPI-A is a glycolipid that can be attached to the C-terminus of proteins during post-translational modification. As a result, the affected HSPC are deficient in the surface display of GPI-anchored protein, generating thereby a phenotype thought to be responsible for clonal advantage and expansion. Among many GPI-anchored proteins, CD55 and CD59 (involved in inhibition of activated C3 and C5 respectively), similar to other GPI-anchored proteins, are strongly associated with lipid rafts. PNH-affected HSPCs, which are missing GPI-A, produce red blood cells, leucocytes and platelets that are susceptible to complement-mediated lysis 9–12. The haemolytic episodes may occur late at night when the CC is activated because of a decrease in blood pH or during infections 9–12. In addition, we have previously demonstrated that a lack of PIG-A on the surface of K-562 cells results in defective lipid raft function and may change the response of these cells to pro-inflammatory cytokines 13.

Since both CXCR4 and VLA-4 depend for their optimal function on their presence within lipid rafts 14, we have been suggested that the PIG-A mutation seen in PNH patients may result in defective retention of PNH-affected HSPCs in BM. In our previous short communication, we demonstrated that a majority of CD34^+^ HSPCs circulating under steady-state conditions in PB in PNH patients belong to a defective clone 15. These cells do not bind a fluorescent variant of aerolysin (FLAER), which identifies the presence of GPI-A on the cell surface in FACS analysis, and thus are FLAER^−^ 15. We also previously demonstrated that FLAER^−^ cells circulating in PB have defective adhesion 15.

In the current study, we expand on our previous observations and provide further evidence for defective retention of PNH HSPCs in BM. These studies have been performed on: (*i*) FACS-purified normal human FLAER^+^ and PNH-affected FLAER^−^ cells isolated directly from BM; (*ii*) Jurkat GPI-A^−^ and GPI-A^+^ cells and (*iii*) normal mice transplanted with BM cells lacking endogenous CC inhibitors (CD55^−/−^ CD59^−/−^) but that express GPI-A anchor normally on the cell surface.

Here, we show for the first time that BM-derived CD34^+^ PNH cells similarly as circulating in blood CD34^+^ PNH cells 15 show a defect in incorporation of not only CXCR4 but also VLA-4 into membrane lipid rafts, respond weakly to SDF-1 stimulation and show impaired adhesion and chemotaxis in response to an SDF-1 gradient. Data obtained for primary patient cells were reproduced by using human GPI-A^−^ Jurkat T-cell line. Moreover, to exclude the potential involvement of CD55 and CD59 antigens in the expansion of HSPCs in BM, we co-transplanted wild-type (WT) mice with CD55^−/−^ CD59^−/−^ BM cells mixed with WT marrow cells in different ratios and observed no preferential expansion of CD55^−/−^ CD59^−/−^ cells over WT cells in transplanted hosts in which GPI-A was present on HSPCs.

Based on these results, our data explain the expansion of PNH-mutated HSPCs in BM in a novel way. Specifically, since these cells have defective adhesion due to a defect in lipid raft formation, they are more mobile in the BM microenvironment and over time expand and outcompete normal HSPCs from their niches. Furthermore, S1P is a major chemotactic factor for HSPCs and its level is high in PB, both under steady-state conditions and during complement-mediated haemolysis. This understanding may also help direct therapeutic strategies for this disease in the future. For instance, to prevent uncontrolled mobility of PNH-affected HSPCs and their subsequent egress from their niches, once could also consider using molecules that attenuate the function of the S1P–S1P receptor axis along with CC inhibitors (*e.g*. eculizumab) 10,16,6.

## Material and methods

After obtaining informed consent, samples of BM cells were aspirated from eight patients diagnosed with PNH and injected into tubes containing anti-coagulant. Low-density bone marrow mononuclear cells (BMMNCs) were isolated after Ficoll/Paque gradient centrifugation. All studies using patient cells were approved by the appropriate research ethics boards at participating institutions.

### FACS sorting of FLAER^+^ and FLAER^−^ cells as well as CD34^+^ FLAER^+^ and CD34^+^ FLAER^−^ cells

Samples of BM were collected from PNH patients and control subjects. The full population of BM MNCs was obtained after density-gradient centrifugation using Ficoll-Paque PLUS reagent (GE Healthcare, Buckinghamshire, UK). A single-cell suspension was stained with mouse anti-human CD34 antibody (APC; BD Bioscience, San Jose, CA, USA) and fluorescently labelled aerolysin (FLAER, Alexa Fluor® 488; Cedarlane Laboratories, Burlington, NC, USA). 18 Staining was carried out on ice for 30 min. Cells were washed and sorted using a MoFlo™ XDP cell sorter (Beckman Coulter, Brea, CA, USA). Sorted populations of FLAER^−^ and FLAER^+^ cells were used for chemotaxis and adhesion assays, and the populations of FLAER^−^/CD34^+^ and FLAER^+^/CD34^+^ cells used for immunocytochemistry staining.

### Cell adhesion studies

The wells of 96-well plates were coated with fibronectin (10 μg/ml) overnight at 4°C and blocked with 0.5% bovine serum albumin (BSA) for 2 hrs before the experiment. After inducing quiescence with 0.5% BSA in RPMI cell suspensions (5 × 10^3^/100 μl), FLAER^−^ and FLAER^+^ or Jurkat GPI^+^ and Jurkat GPI^−^ cells were added directly to the fibronectin-coated 96-well plates and incubated for 5, 10 or 15 min. at 37°C. Following incubation, the plates were vigorously washed three times to remove non-adherent cells, and the number of adherent cells was counted using an inverted microscope. In addition, an adhesion assay was performed after SDF-1 (10 or 100 ng/ml) stimulation.

### Cell migration studies

The Transwell migration assay was performed as described elsewhere 5,18. Briefly, unless otherwise indicated, RBC-lysed samples containing BMMNCs from C57BL/6 mice were resuspended in assay medium (RPMI containing 0.5% BSA) and equilibrated for 10 min. at 37°C. An assay medium (650 μl) containing test reagents was added to the lower chambers of a Transwell plate (Corning Costar, Cambridge, MA, USA). Aliquots of cell suspension (1 × 10^6^ cells/100 μl) were loaded onto the upper chambers with 5-μm-pore filters, then incubated for 3 hrs (37°C, 5% CO_2_). Cells from the lower chambers were scored using FACS analysis for migration of BMMNCs. The results are expressed as fold increase, *i.e*., the ratio of the number of cells that migrated towards the test reagents to the number of cells that migrated towards medium alone. In some experiments, migrated cells harvested from the lower chambers were plated in colony-forming assays, for which, the following concentrations of growth factors were used: GM-CSF (25 ng/ml) and IL-3 (10 ng/ml).

### Cell signalling studies

Cells were kept in RPMI containing 0.5% BSA overnight in an incubator to achieve quiescence, stimulated with SDF-1 (300, 100, or 30 ng/ml), and then lysed for 20 min. on ice with the RIPA lysis buffer system (Santa Cruz Biotechnology, Dallas, TX, USA), containing protease and phosphatase inhibitors (Santa Cruz Biotechnology) as described 5,18. The concentrations of extracted proteins were measured with the BCA protein assay kit, according to the manufacturer’s instructions (Thermo Scientific, Rockford, IL, USA), and equal amounts of protein were separated and analysed for phosphorylation of p44/42 MAPK and AKT (Ser473). Equal loading in the lanes was evaluated by stripping the blots and reprobing with antibodies for p44/42 MAPK and AKT. All phosphospecific antibodies were purchased from Cell Signaling Technology Inc. (Danvers, MA, USA). The membranes were developed with Amersham ECL Western Blotting Detection reagents and exposed to Amersham Hyperfilm (GE Healthcare).

### Lipid raft formation analysis

To visualize lipid rafts, a confocal microscopy assay was performed as previously described 5,14,15. Briefly, cells were plated on poly-L-lysine-coated plates overnight, then incubated with LL-37 (2.5 μg/ml) and SDF-1 (50 ng/ml) for 1 hr, then washed and fixed in 3.7% paraformaldehyde. Cholera toxin subunit B conjugated with fluorescein isothiocyanate (FITC; Sigma-Aldrich, Saint Louis, MO, USA) was applied to detect the ganglioside GM1, and mouse monoclonal anti-hCXCR4 IgG antibody (R&D Systems, Minneapolis, MN, USA) and Alexa Fluor 594 goat antimouse IgG antibody (Invitrogen, Carlsbad, CA, USA) were applied to detect CXCR4. VLA-4 was stained with monoclonal rat antimouse integrin alpha 4 antibody (CD49d; EMD Millipore, Billerica, MA, USA) and Alexa Fluor 594. Stained cells were examined, and images were generated using a FlouView FV1000 laser-scanning confocal microscope (Olympus America Inc., Center Valley, PA, USA).

### Actin polymerization

Actin polymerization was determined as previously described 14. Briefly, cells were resuspended in RPMI containing 0.5% BSA at a concentration of 1 × 10^6^ cells/ml, incubated with SDF-1 and/or LL37, then fixed and permeablized. Alexa 488 phalloidin (Invitrogen) was added to visualize F-actin. Mean fluorescence intensity was measured with an LSR II flow cytometer (Becton Dickinson, Mountain View, CA, USA). The relative fluorescence change was normalized by the change for unstimulated cells.

### BM transplants in mice

WT mice were lethally irradiated and 24 hrs later transplanted with WT and double knockout CD55^−/−^ CD59^−/−^ cells 19 in a 1:1 ratio. Before transplant, the level of knockout of CD55 and CD59 was confirmed by FACS analysis. Four months after transplant, PB was collected to recheck the knockout levels. We used the following antibodies for FACS staining: PE hamster antimouse CD55 (BD, Franklin Lakes, MJ, USA) and FITC-conjugated monoclonal antibody to mouse CD59a glycoprotein (HyCult Biotech, Plymouth Meeting, PA, USA).

### Statistical analysis

Arithmetic means and standard deviations were calculated using Instat 1.14 software (Graphpad, San Diego, CA, USA). Statistical significance was defined as *P* < 0.05. Data were analysed using Student’s *t*-test for unpaired and paired samples, as appropriate.

## Results

### BM-isolated FLAER-negative cells show defective adhesion, chemotaxis and signalling responsiveness to SDF-1

**Figure 1:**
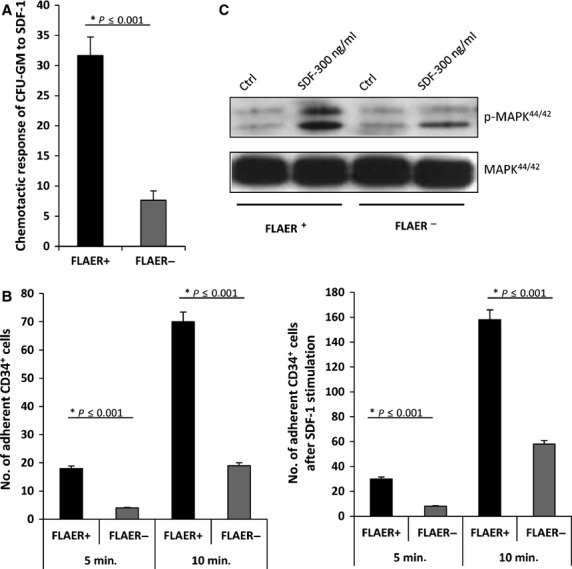
Human BM-purified FLAER^−^ cells show impaired chemotaxis, adhesion and responsiveness to SDF-1. (A) FACS-sorted FLAER^−^ and FLAER^+^ BMMNCs were tested for their chemotaxis in response to an SDF-1 gradient and cells that migrated to the lower chambers were plated in methylcellulose cultures to growth CFU-GM colonies. Data from four separate experiments are pooled together, *P < 0.001. (B) Normal CD34^+^ FLAER^+^ and PNH-affected CD34^+^ FLAER^−^ cells sorted from BMMNCs were tested in 5- and 10-min. assays for adhesion to fibronectin (left panel) or to SDF-1 (right panel) immobilized on the bottom of the plates. The number of adherent cells in both cell populations is indicated, and data from four separate experiments are pooled together. *P < 0.001 compared with untreated controls. (C). Phosphorylation of p42/44 MAPK and AKT in FACS-sorted FLAER^−^ and FLAER^+^ BMMNCs. Cells were made quiescent and stimulated for 10 min. with SDF-1 (300 ng/ml). One representative blot out of two is shown.

Fluorescently labelled aerolysin detects the presence of GPI-A on the cell surface 17. Thus, BMMNCs from PNH patients were sorted into FLAER^−^ and normal FLAER^+^ cells. These cells were subsequently tested in various assays to assess their chemotaxis, adhesion and responsiveness in signalling assays to stromal-derived factor 1 (SDF-1) stimulation (Fig.1).

Paroxysmal nocturnal haemoglobinuria-affected FLAER^−^ clonogenic progenitors (CFU-GM) responded very weakly to a SDF-1 gradient in a Transwell chemotaxis assay (Fig.1A). At the same time, compared with normal FLAER^+^ BMMNCs, FLAER^−^ BMMNCs had defective adhesion to fibronectin, both in short (5 min.) and prolonged (10 min.) adhesion assays (Fig.1B). Both adhesion and chemotaxis studies were corroborated by SDF-1 signalling studies and FLAER^−^ BMMNCs responded very weakly to stimulation by SDF-1 compared with normal FLAER^+^ BMMNCs (Fig.1C).

### BM-isolated CD34^+^ FLAER^−^ cells show defective adhesion and incorporation of CXCR4 and VLA-4 into membrane lipid rafts

Next, we sorted PNH-affected CD34^+^ FLAER^−^ and normal, healthy CD34^+^ FLAER^+^ BMMNCs by FACS (*n* = 8). As expected, we observed higher numbers of BM FLAER^−^ CD34^+^ cells than normal FLAER^+^ CD34^+^ cells in PNH patients (3.3 ± 0.7% *versus* 0.8 ± 0.5%, respectively).

**Figure 2:**
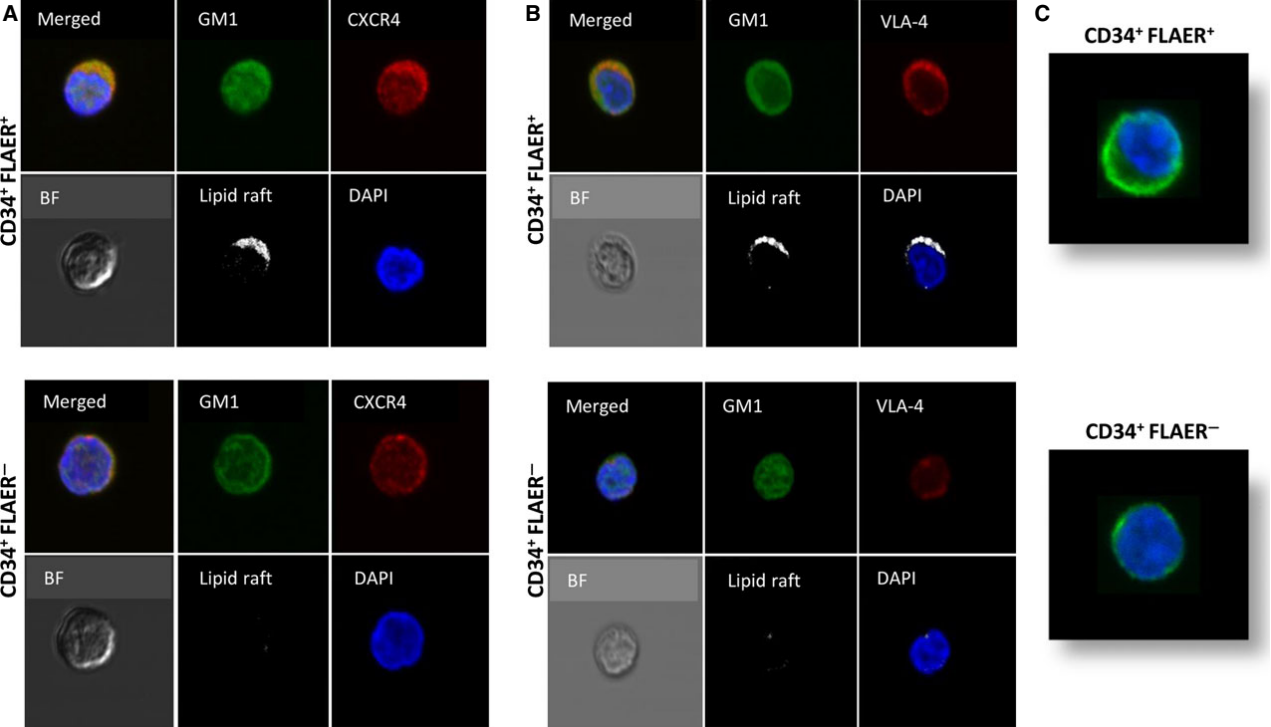
Defective adhesiveness and lipid raft formation in BM-derived CD34^+^ FLAER^−^ cells (A and B). Representative images of CD34^+^ FLAER^+^ (normal) and CD34^+^ FLAER^−^ (PNH) cells sorted from BM, stimulated by LL-37 (2.5 μg/ml), stained with cholera toxin subunit B (a lipid raft marker) conjugated with FITC, rabbit anti-hCXCR4 antibody with anti-rabbit Alexa Fluor 594, rat antimouse VLA-4 with Alexa Fluor 594, and evaluated by confocal microscopy for formation of membrane lipid rafts. White areas indicate colocalization of CXCR4 (A) and VLA-4 (B) in membrane lipid rafts. It can be seen that lipid rafts were formed in CD34^+^ FLAER^+^ (normal), but not in CD34^+^ FLAER^−^ (PNH) cells. The experiment was repeated with cells from three different patients, with similar results. (C). When plated in polylysine-coated dishes, CD34^+^ FLAER^−^ cells, in contrast to normal healthy CD34^+^ FLAER^+^ cells, display a defect in actin polymerization. The experiment was repeated three times employing cells from different patients, with similar results.

Since we found that CD34^+^ FLAER^−^ cells (Fig.1B), like FLAER^−^ BMMNCs (data not shown), have defective 5-min. and 15-min. adhesion to both fibronectin- and SDF-1-coated plates and while adhesion to SDF-1 is CXCR4-dependent, and adhesion to fibronectin is mostly VLA-4-dependent, we investigated by confocal analysis whether both receptors are incorporated into lipid rafts in patient BM-purified CD34^+^ FLAER^−^ cells. Lipid raft formation was analysed in the presence of cationic peptide LL-37, which promotes lipid raft formation on the surface of hematopoietic cells 20,21. We found that CD34^+^ FLAER^−^ cells have a defect in lipid raft formation compared with normal CD34^+^ FLAER^+^ cells, and neither CXCR4 nor VLA-4 are detected in lipid rafts (Fig.2A and B). At the same time, we observed a defect in actin polymerization in CD34^+^ FLAER^−^ cells compared with healthy CD34^+^ FLAER^+^ cells (Fig.2C).

### GPI-A^−^ Jurkat cells show defective spontaneous and SDF-1-stimulated adhesion to fibronectin as well as defective SDF-1 signalling, and they do not incorporate CXCR4 and VLA-4 into lipid rafts

**Figure 3:**
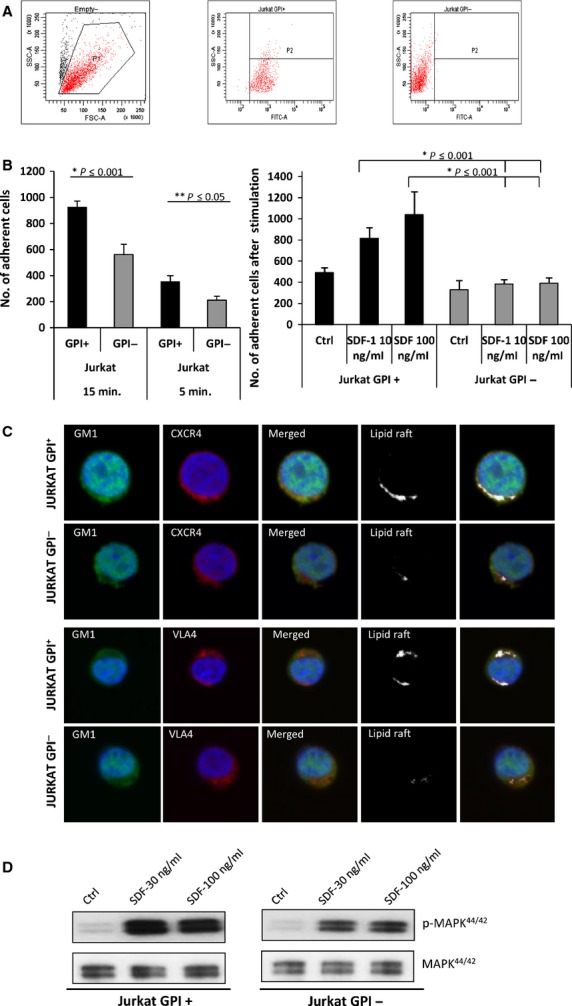
Defective SDF-1 responsiveness of GPI-A-deficient human Jurkat cells. (A). Binding of FLAER to GPI-A-deficient and normal Jurkat cells. One representative staining out of three is shown. (B). Jurkat GPI-A-deficient cells show defective spontaneous (left panel) and SDF-1-stimulated (right panel) adhesion to fibronectin-coated plates. Data from four separate experiments are pooled together. *P < 0.01 compared with normal Jurkat cells. (C). Representative images of FLAER^+^ (normal) and FLAER^−^ human Jurkat cells stimulated by LL-37 (2.5 μg/ml), stained with cholera toxin subunit B (a lipid raft marker) conjugated with FITC, rabbit anti-hCXCR4 antibody with anti-rabbit Alexa Fluor 594, and rat antimouse VLA-4 (EMD Millipore) with Alexa Fluor 594, and evaluated by confocal microscopy for formation of membrane lipid rafts. White areas indicate colocalization of CXCR4 and VLA-4 in membrane lipid rafts. It can be seen that lipid rafts were formed in normal FLAER^+^ but not in FLAER^−^ Jurkat cells. The experiment was repeated three independent times with similar results. (D). Jurkat GPI-A^+^ and GPI-A^−^ cells were starved in an incubator overnight in RPMI containing 0.5% BSA, stimulated with SDF-1 (30 or 100 ng/ml) for 5 min., and evaluated using Western blot assay. Experiments were repeated independently four times with similar results. A representative western blot is shown.

Next, we performed similar experiments with GPI-A-deficient and GPI-A-expressing Jurkat human lymphocytic T-cell lines 13. GPA-I-A^−/−^ Jurkat cells demonstrated a lack of FLAER binding (Fig.3A), and by employing adhesion assays, we observed that these cells show defective spontaneous 5 and 15 min. adhesion to fibronectin (Fig.3B, left panel), which also remained defective after pre-treatment of cells with SDF-1 (0–100 ng/ml, Fig.3B, right panel). FLAER^−^ Jurkat cells, like normal BM-purified CD34^+^ FLAER^−^ cells, did not incorporate CXCR4 and VLA-4 into membrane lipid rafts (Fig.3C). Finally, GPI-A^−^ Jurkat cells demonstrated a decrease in phosphorylation of p42/44 MAPK in response to SDF-1 (Fig.3D).

### Murine BM-derived CD55^−/−^ CD59^−/−^ cells that properly express GPI-A show normal adhesion and chemotaxis in response to SDF-1 and do not outcompete wild-type BM cells after transplantation into normal recipients

**Figure 4:**
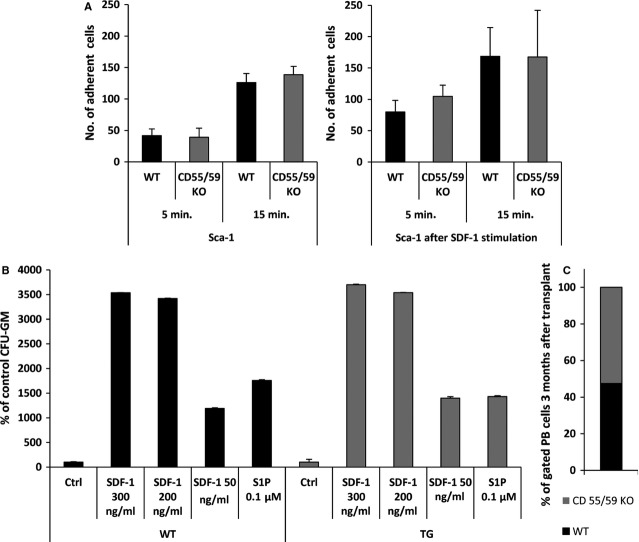
BM cells from CD55^−/−^ CD59^−/−^ mice have normal adhesion and chemotaxis and do not expand in transplanted wild-type control animals (A). BM Sca-1^+^ cells from CD55^−/−^ CD59^−/−^ mice were tested in short (5 min.) and prolonged (15 min.) fibronectin adhesion assays with or without stimulation by SDF-1. Data are pooled from experiments on BM cells isolated from three different mice (B). BM Sca-1^+^ cells from CD55^−/−^ CD59^−/−^ mice were tested in a Transwell chemotaxis assay in response to different SDF-1 gradients (50–300 ng/ml). Data are pooled for experiments on BM cells isolated from three different mice. (C). Normal wild-type mice were transplanted with BM from CD55^−/−^ CD59^−/−^ mice mixed in a 1:1 ratio with BM from wild-type littermates. These animals maintained ∼1:1 chimerism. Shown are FACS data performed on BM pooled from six recipient mice.

Human PNH cells, which lack GPI-A and therefore do not express the complement inhibitors CD55 and CD59 on their cell surface, expand over time in BM. To dissect the potential involvement of the absence of CD55 and CD59 in this expansion, we isolated BM from CD55^−/−^ CD59^−/−^ mice 19 and tested these cells in adhesion and chemotaxis assays. Murine Sca-1^+^ CD55^−/−^ CD59^−/−^ cells displayed normal adhesion to fibronectin-coated plates with or without SDF-1 pre-incubation (Fig.4A, left and right panels, respectively) and showed normal chemotaxis in response to an SDF-1 gradient in a Transwell assay compared with BM cells isolated from normal littermates (Fig.4B).

On the basis of the observation that PNH-affected cells expand in patient BM over time, we transplanted BM cells from CD55^−/−^ CD59^−/−^ mice and normal WT BM cells CD55^+/+^ CD59^+/+^ mixed in different ratios (1:9, 1:3, 1:1, 3:1 and 9:1) into normal WT mice. Six months after transplantation, we analysed the percentage of chimerism in PB, BM and spleen of recipient mice and did not observe significant changes in the ratio of transplanted mutant to normal cells in chimeric mice, who retained 1:1 chimerism (Fig.4C). This finding further supports the conclusion that a defect in lipid raft formation, and not the absence of CD55 and CD59 antigens on the cell surface, is responsible for the expansion of PNH-affected cells in the BM microenvironment.

## Discussion

We report here that BM-purified PNH-affected CD34^+^ HSPCs similarly as PNH-affected CD34^+^ HSPCs circulating in blood 15 have defective adhesion, chemotaxis and responsiveness to an SDF-1 gradient and show a defect in incorporation of CXCR4 and VLA-4 into membrane lipid rafts. Moreover, we reproduced similar results with GPI-A-defective Jurkat cells. Furthermore, by employing CD55^−/−^ CD59^−/−^ BMMNCs and WT BMMNC radiation chimeras, we eliminated the potential involvement of the lack of expression of CD55/CD59 in the clonal expansion of CD55^−/−^ CD59^−/−^ cells, in which, the proper function of GPI-A is preserved.

PNH is an uncommon acquired haemolytic anaemia that, as a result of GPI-A deficiency, often manifests with haemoglobinuria, abdominal pain, smooth muscle dystonia, fatigue and thrombosis. PNH can arise *de novo* or in conjunction with aplastic anaemia. The PIG-A gene is located on the X chromosome, and because of inactivation of one of the X chromosomes in somatic cells, the ratio of the incidence of PNH between females and males is ∼1:1 9–11. Since GPI-A is neither an oncogene nor an anti-oncogene, PNH-affected HSPCs expansion in BM over time is poorly understood. Over the past several years, several theories have been proposed to explain clonal expansion of PNH cells, including: (*i*) PNH clones escape immune attack; (*ii*) a second unidentified mutation gives PNH cells a growth/expansion advantage; (*iii*) PNH mutants have a greater resistance to apoptosis than normal cells and (*iv*) a deficiency of GPI-A-linked UL16-binding proteins involved in the elimination of cytomegalovirus.

Furthermore, it is most likely that normal healthy individuals harbour dormant PNH clones in BM as a result of mutations acquired during life. Depending on circumstances, these clones may remain quiescent or expand to become clinically relevant, as seen in aplastic anaemia patients after endogenous stimulation of haematopoiesis 9–11. We have previously demonstrated in previous study that human PIG-A-deficient K-562 cells have defective membrane lipid rafts 13 and that membrane lipid rafts are essential for optimal interaction of CXCR4 with its ligand, SDF-1 14. We also observed in PNH patients that FLAER^−^ CD34^+^ cells are preferentially mobilized from BM into PB 15. In our current study, we extended these observations and demonstrated that PNH-affected FLAER^−^ cells isolated from BM have defective adhesion, chemotaxis and responsiveness to SDF-1 gradients.

Membrane lipid rafts are important not only for proper function of CXCR4 14 but also for proper interaction between integrin receptors 21 expressed on HSPCs and their corresponding ligands in stem cell niches. One of the crucial integrins that plays a role in retention of HSPCs in the BM microenvironment is VLA-4, which is expressed on HSPCs and interacts with VCAM-1 expressed in the BM niche 1–4. The current study showed that BM-derived FLAER^−^ cells have defective adhesion to fibronectin, a VLA-4 ligand. Importantly, by employing confocal microscope analysis we demonstrated defective incorporation of both CXCR4 and VLA-4 into membrane lipid rafts of CD34^+^ FLAER^−^ cells isolated from PNH BM. Since membrane lipid rafts facilitate interaction of CXCR4 and VLA-4 with Rho and Rac GTPases 21, which are components of the adhesion and migration signalling pathways, this may explain why BM-purified PNH cells show defective adhesion and migratory responses to an SDF-1 gradient.

On the basis of our previously published 13,15 and current data, we propose a novel explanation for why, over time, PNH-affected clones expand and become dominant over normal HSPCs in BM. We propose that PNH cells have defective adhesion because of a defect in lipid raft formation, they are more mobile in the BM microenvironment and over time expand and out-compete normal HSPCs from their niches. This explanation is based on: (*i*) the pivotal role of membrane lipid rafts in the function of CXCR4 and VLA-4 in the active retention of HSPCs in BM; (*ii*) the fact that PNH-affected cells do not properly incorporate CXCR4 and VLA-4 into membrane lipid rafts; (*iii*) the presence of a continuous S1P gradient in PB for HSPCs actively attached in BM niches; (*iv*) the crucial role of activated complement cascade in mobilization of HSPCs and (*v*) the fact that S1P, which is highly enriched in erythrocytes, increases in PB during complement-mediated erythrocyte haemolysis and platelet activation 16,6. Our published data on the role of S1P in the migration of HSPCs indicate that S1P receptors (S1P_1_ and S1P_3_) are not associated with membrane lipid rafts. Thus, PNH-affected HSPCs may respond normally to S1P, in contrast to an SDF-1 gradient.

Taking all these considerations together, we propose that defective adhesion of HSPCs in BM plays an important role in the pathogenesis of PNH and that a PNH clone that is highly mobile and responsive to S1P may with time out-compete normal HSPCs for their niches. Thus, a defect in a gene involved in adhesion and the attenuated interaction of HSPCs with their BM niche, and not the involvement of an oncogene or anti-oncogene mutation, may lead to proliferation of defective PNH cells in BM and their domination over normal HSPCs. Given the heterogeneity in clone sizes in PNH patients, however, a key question still remains to be answered: once a PNH clone expands by the mechanism proposed above, what determines its eventual size (*e.g*. 1% *versus* 95%)? The answer to this question may also help us understand spontaneous remissions or clone size reductions that have been reported in up to 15% of PNH cases 11. The different clone sizes may represent various phases during the course of events described above; however, it has been reported that most PNH patients retain the same clone size over time 12. The precise factors that determine the proliferation rate of PNH clones and why this stops at different levels in different patients remain unclear.

HSPCs are mobilized in several other haemolytic syndromes in addition to PNH, including sickle cell anaemia 22; however, HSPC mobilization in sickle cell anaemia is not as extensive as in PNH, because HSPCs in sickle cell anaemia do not have a defect in lipid raft formation and may be normally retained in BM niches despite a significant increase in the level of S1P in PB during haemolytic crisis. In support of this notion, we recently demonstrated in a phenylhydrazine-induced haemolysis murine model that an increase in S1P level without simultaneous blockade of the SDF-1–CXCR4 interaction in the BM niche had very little effect on the egress of HSPCs from BM into PB 23. Thus, a lipid raft formation defect and defective lipid raft-mediated retention of HSPCs in BM niches seem to be crucial for enhanced mobility of PNH-affected HSPCs, both in response to a steady-state level and to an elevated level of S1P in PB during CC-initiated haemolytic incidents.

It has been demonstrated that the CC shows a circadian rhythm of activation, with a peak during sleep because of a drop in blood pH 24 that explains the onset of haemolysis and haemoglobinuria late at night. Moreover, since the CC is activated during infections, the role of the CC in PNH explains the exacerbation of PNH symptoms patients suffering from infection 9–12. Since membrane attack complex C5b-C9 (MAC) is responsible for erythrocyte lysis in PNH patients, this also explains the effectiveness of CC inhibitors, such as eculizumab 9 or compstatin, in treatment of this disorder 25.

In conclusion, our data shed new light on the pathogenesis of PNH and explain why PNH clones expand over time and outcompete normal HSPCs in BM in PNH patients. Better understanding of this process may eventually lead to more effective therapies in these patients. We propose that to prevent uncontrolled motility of PNH-affected HSPCs and their egress from BM niches, the use of S1P–S1P receptor axis inhibitors (*e.g*. fingolimod) 7 with CC inhibitors may be considered for treatment of PNH 9,25. Finally, it has been reported that PNH may develop as result of immunosuppressive therapy in patient with aplastic anaemia, what suggests that PNH clone may have growth advantage not only over normal HSPCs but also over aplastic clone of cells 26. This interesting phenomenon, however, requires further studies.
